# Colony Collapse Disorder in context

**DOI:** 10.1002/bies.201000075

**Published:** 2010-10

**Authors:** Geoffrey R Williams, David R Tarpy, Dennis vanEngelsdorp, Marie-Pierre Chauzat, Diana L Cox-Foster, Keith S Delaplane, Peter Neumann, Jeffery S Pettis, Richard E L Rogers, Dave Shutler

**Affiliations:** Department of Biology, Dalhousie UniversityHalifax, NS, Canada; Department of Biology, Acadia UniversityWolfville, NS, Canada; Department of Entomology, North Carolina State UniversityRaleigh, NC, USA; Department of Entomology, The Pennsylvania State University, University ParkPA, USA; French Food Safety Agency (AFSSA)Sophia-Antipolis, France; Department of Entomology, University of GeorgiaAthens, GA, USA; Swiss Bee Research Centre, Agroscope Liebefeld-Posieux Research Station ALPBern, Switzerland; Department of Zoology and Entomology, Rhodes UniversityGrahamstown, South Africa; United States Department of Agriculture, Agricultural Research ServiceBeltsville, MD, USA; Ecotoxicology, Bayer CropScienceResearch Triangle Park, NC, USA

Although most of humanity relies upon foods that do not require animal pollination 1, production of 39 of the world's 57 most important monoculture crops still benefits from this ecosystem service 2. Western honey bees (*Apis mellifera*) are undoubtedly the single-most valuable animal pollinators to agriculture because they can be easily maintained and transported to pollinator-dependent crops. Yet, despite an almost 50% increase in world honey bee stocks over the last century, beekeepers have not kept pace with the >300% increase in pollinator-dependent crops 3. This has led to great uncertainty surrounding the recent large-scale die-offs of honey bees around the world, and has sparked enormous interest from both scientists and the general public.

Although sharp regional declines in honey bee populations have occurred in the past, such as the so-called unexplainable “Isle of Wight” disease in the early 1900s 4, the magnitude and velocity of these recent declines are likely unprecedented. Often in the media (*e.g*. “Mobile phones responsible for disappearance of honey bee,” available at http://www.telegraph.co.uk/), and sometimes in the scientific literature (*e.g*. 5), these losses are inappropriately equated with “Colony Collapse Disorder” or CCD, which is characterized by the rapid disappearance of adult bees from colonies containing brood and food stores but lacking damaging levels of parasitic *Varroa destructor* mites or *Nosema* microsporidians 6.

Although, we agree that CCD is indeed a significant cause for concern, we believe that it is imperative to appropriately place CCD within the greater context of other honey bee morbidities occurring worldwide. In many cases, these morbidities can be explained by known parasites or beekeeper management issues. One example is the devastation caused by beekeepers' inability to control *V. destructor*, which not only feeds on host haemolymph and weakens host immunity but also vectors a variety of viruses 7. In other cases, however, these morbidities are genuinely unexplainable, including those attributed to CCD *sensu stricto* 6. In recent winters, colony mortality in Europe has averaged ∼20% (ranging from 1.8 to 53% among countries), with starvation and parasites believed to be the main contributors (“Proceedings of the 4th COLOSS Conference, Zagreb, Croatia, 3-4 March 2009”, available at http://www.coloss.org/publications). Colony mortality during the 2006/2007, 2007/2008, and 2008/2009 winters in the US, the only country where CCD has been documented *sensu stricto*, was 32% 8, 36% 9, and 29% 10, respectively. During the winter of 2008/2009, ∼10% of the 2.3 million managed honey bee colonies in the US died with “CCD-like symptoms”, and US beekeepers self-diagnosed CCD as only the 8th most important contributor to colony mortality, behind starvation, queen-related issues, and parasites 10. The point is, honey bees die from many things. We must be careful to not synonymize CCD with all honey bee losses.

There is a growing consensus that colony mortality is the product of multiple factors, both known and unknown, acting singly or in combination 11, 12. Considering the reliance that modern agriculture places on honey bees for pollination, coordinated efforts, such as those of CANPOLIN (Canadian Pollination Initiative, http://www.uoguelph.ca/canpolin), COLOSS (Prevention of Honeybee Colony Losses, http://www.coloss.org/), and the US Department of Agriculture's Areawide and Managed Pollinator CAP (Coordinated Agricultural Project) 13, are urgently needed to understand and mitigate these losses. The first step in these efforts should be to objectively discriminate among types of colony mortality occurring worldwide. This will permit a more informed and appropriate allocation of research efforts into CCD specifically and other causes of mortality in general.

## Abbreviations

CCD: Colony Collapse Disorder
